# Upregulation of Vitamin C Transporter Functional Expression in 5xFAD Mouse Intestine

**DOI:** 10.3390/nu13020617

**Published:** 2021-02-14

**Authors:** Trevor Teafatiller, Christopher W. Heskett, Anshu Agrawal, Jonathan S. Marchant, Janet E. Baulch, Munjal M. Acharya, Veedamali S. Subramanian

**Affiliations:** 1 Department of Medicine, University of California, Irvine, CA 92697, USA; tteafati@hs.uci.edu (T.T.); cheskett@uci.edu (C.W.H.); aagrawal@hs.uci.edu (A.A.); 2 Department of Cell Biology, Neurobiology, and Anatomy, Medical College of Wisconsin, Milwaukee, WI 53226, USA; jmarchant@mcw.edu; 3 Department of Radiation Oncology, University of California, Irvine, CA 92697, USA; jbaulch@hs.uci.edu (J.E.B.); macharya@uci.edu (M.M.A.)

**Keywords:** Vitamin C, transport, Alzheimer’s disease, SVCT1, SVCT2

## Abstract

The process of obtaining ascorbic acid (AA) via intestinal absorption and blood circulation is carrier-mediated utilizing the AA transporters SVCT1 and SVCT2, which are expressed in the intestine and brain (SVCT2 in abundance). AA concentration is decreased in Alzheimer’s disease (AD), but information regarding the status of intestinal AA uptake in the AD is still lacking. We aimed here to understand how AA homeostasis is modulated in a transgenic mouse model (5xFAD) of AD. AA levels in serum from 5xFAD mice were markedly lower than controls. Expression of oxidative stress response genes (glutathione peroxidase 1 (GPX1) and superoxide dismutase 1 (SOD1)) were significantly increased in AD mice jejunum, and this increase was mitigated by AA supplementation. Uptake of AA in the jejunum was upregulated. This increased AA transport was caused by a marked increase in SVCT1 and SVCT2 protein, mRNA, and heterogeneous nuclear RNA (hnRNA) expression. A significant increase in the expression of HNF1α and specific protein 1 (Sp1), which drive SLC23A1 and SLC23A2 promoter activity, respectively, was observed. Expression of hSVCT interacting proteins GRHPR and CLSTN3 were also increased. SVCT2 protein and mRNA expression in the hippocampus of 5xFAD mice was not altered. Together, these investigations reveal adaptive up-regulation of intestinal AA uptake in the 5xFAD mouse model.

## 1. Introduction

Vitamin C (ascorbic acid; AA) is important for maintaining normal cellular function and overall health as it acts as an essential cofactor for several key enzymatic reactions [1,2]. Maintaining vitamin C body homeostasis seems to protect against age-related cognitive decline and Alzheimer’s disease (AD) [3]. Deficiency of vitamin C is implicated in cognitive dysfunction and accelerates β-amyloid accumulation and deposition in AD [4,5,6,7]. Vitamin C can also play a role in immunosenescence and inflammation, the trademarks of biological aging [6]. The brain is a highly metabolically active organ that is vulnerable to free radical damage and oxidative stress, which may play a pivotal part in AD pathogenesis. It is well established that vitamin C is a first-line antioxidant defense that neutralizes reactive oxygen species (ROS) by promoting the regeneration of other endogenous antioxidants [6]. Plasma vitamin C levels in AD patients are significantly lower than that of normal individuals, which may correlate with adverse health outcomes [6,8,9,10]. Supplementation of vitamin C to patients with AD has been shown to have beneficial effects including reducing oxidative stress, mitigating disorganization of chromatin and lessening excessive secretion of inflammatory factors [6]. Thus, studies intended at understanding the underlying mechanisms involved in maintaining and regulating vitamin C body homeostasis are crucial for designing effective approaches for optimizing vitamin C levels in chronic neurodegenerative conditions, including AD, dementia and aging, where suboptimal vitamin C levels may be detrimental.

Human cells cannot produce vitamin C endogenously; hence, this vitamin is supplied from intestinal absorption and obtained from the circulation (i.e., blood, cerebrospinal fluid (CSF)). Vitamin C uptake occurs through a Na^+^-dependent carrier-mediated process via human Sodium-dependent Vitamin C Transporters in the intestine (hSVCT1 and hSVCT2, the products of the *SLC23A1* and *SLC23A2* genes, respectively) and brain (hSVCT2) [9,11,12]. Despite endogenous synthesis of vitamin C in mice, sufficient dietary vitamin C is still needed to fulfill metabolic requirements as demonstrated using knockout mouse models [13,14]. Accumulation of vitamin C in the CSF occurs by active transport at the choroid plexus through the SVCT2 and this accumulation of vitamin C is in equilibrium with the extracellular fluid (ECF) of the brain by diffusion [11,15]. Further, vitamin C is transported into neuronal cells through SVCT2 [15]. The strong expression of hSVCT2 in the brain and the high retention of vitamin C levels in the central nervous system (CNS) [9,11] suggest essential metabolic roles for vitamin C in neuronal health. Studies on the molecular physiology and pathophysiological dysregulation of the AA uptake process are important to understand the link between brain and intestinal transport systems to gain insight into options for vitamin C and disease management. 

Our investigation aims to understand how the intestinal vitamin C transport process is regulated in an accelerated transgenic mouse model that expresses five Familial Alzheimer’s Disease (5xFAD) mutations [16]. At present, very limited information is available that describes the effect of vitamin C insufficiency/deficiency on intestinal and CNS AA uptake processes in AD. Addressing this issue is crucial to better understand adaptive regulation of the gut and brain AA uptake. This study provides valuable information regarding the intestinal and CNS vitamin C transporter process under normal and pathophysiological conditions. Further, these data will help the development of effective strategies to optimize intestinal and neuronal vitamin C body homeostasis, especially in conditions of vitamin C deficiency relevant to CNS pathologies.

## 2. Materials and Methods

### 2.1. Reagents

^14^C-Ascorbic acid (Cat# NEC1460; specific activity of 2.8 mCi/mmol; radiochemical purity > 97%) was obtained from PerkinElmer (Boston, MA, USA). All molecular biological and biochemical components were from Sigma (St. Louis, MO, USA) or Thermo Fisher Scientific (Carlsbad, CA, USA). DNA oligonucleotide primers used in this study were procured from Integrated DNA Technologies (San Diego, CA, USA). Secondary antibodies and blocking buffer for western blotting were purchased from LI-COR Biosciences (Lincoln, NE, USA). 

### 2.2. Animals 

The well-characterized 5xFAD mouse model of AD overexpresses both mutant human amyloid precursor protein (APP) (695) with the Swedish (K670N, M671L), Florida (I716V), and London (V717I) Familial Alzheimer’s Disease (FAD) mutations and human PS1 harboring two FAD mutations, M146L and L286V (MMRRC strain: 03448) [16]. These transgenic mice represent an accelerated AD model, developing AD pathologies by 2 months of age and reductions in synaptophysin, neuron loss and memory impairments that become evident by 5–6 months of age [16]. Twelve-month-old male 5xFAD mice and their wild-type (WT) littermate controls were maintained in standard housing conditions as described before [17] and provided ad libitum access to standard rodent chow (Envigo Teklad 2020x) and water. For AA supplementation studies, mice were provided 3.3g/l of AA in drinking water for 1 week and then used for oxidative stress response genes expression studies. Mice were anesthetized as described before [17] and the blood, hippocampus, and jejunum samples were collected for AA level determination, ^14^C-AA uptake assay, molecular biological, and immunohistochemical analysis. Mice experimentation procedures were performed in accordance with the National Institutes of Health (NIH) rules and permitted by the University of California Irvine, Institutional Animal Care, and Use Committee (IACUC). 

### 2.3. AA Determination in Mice Serum 

The 5xFAD and WT littermate mice blood samples (4 animals/group) were collected and centrifuged to separate the serum and the level of AA in the serum was determined using the AA Colorimetric Assay Kit II (FRASC) (Cat# K671; BioVision, Inc., Milpitas, CA). In this assay, Fe^3+^ is reduced to Fe^2+^ by any antioxidants present. The ferrous iron is chelated with a colorimetric probe to produce a product with a strong absorbance band that can be monitored between 545–600 nm. The addition of ascorbate oxidase to parallel samples removes any ascorbate present leaving a background value, which is subtracted from the total to give ascorbate content. The assay can detect 0.2 to 20 nmol of AA in samples.

### 2.4. ^14^C-AA Uptake

AA uptake analysis was performed in the jejunum of 5xFAD and WT littermate mice (4–5 animals/group) as described before [12,18]. The animals were sacrificed and the isolated jejunum samples were cut open longitudinally, which were then washed with Krebs Ringer (KR) buffer. The individual samples were subsequently incubated with KR buffer containing labeled (0.1µCi) or labeled plus unlabeled (1mM) AA in a 37°C water bath. Following 7 min of incubation, each sample was lysed using NaOH (1N) and immediately heat treated in an air incubator set to 80°C for 15 min, after which the samples were neutralized by adding HCl (10N). A liquid scintillation counter purchased from Beckman Coulter (Brea, CA, USA) was then used to measure radioactive content. Additionally, Bio-Rad (Hercules, CA, USA) DC Protein Assay reagents were used to determine protein concentrations of the jejunal tissue.

### 2.5. RT-qPCR Analysis

Total RNA from 5xFAD and WT littermate mice jejunum and hippocampus tissues (4–12 animals/group) were isolated using TRIzol reagent (Cat# 15596018; Life Technologies, Carlsbad, CA, USA). The cDNA was synthesized utilizing DNase I (Cat# 18068015; Invitrogen, Carlsbad, CA, USA) and iScript kit reagents (Cat# 1708891; Bio-Rad). SVCT1, SVCT2, GPX1, SOD1, HNF1α, specific protein 1 (Sp1), GRHPR, CLSTN3 and β-actin mRNA expression levels were determined by RT-qPCR using the synthesized cDNA, iQ SYBR Green reagent (Cat# 1708884; Bio-Rad), gene specific primer combinations for SVCT1, SVCT2, GPX1, SOD1, HNF1α, Sp1, GRHPR, CLSTN3 and β-actin (SVCT1: F, 5′-CAGCAGGGACTTCCACCA-3′, R, 5′-CCACACAGGTGAAGATGGTA-3′; SVCT2: F, 5′-AACGGCAGAGCTGTTGGA-3′, R, 5′-GAAAATCGTCAGCATGGCAA-3′; GPX1: F, 5′-CTCTTTACCTTCCTGCGGAA-3′, R, 5′-GGACAGCAGGGTTTCTATGT-3′; SOD1: F, 5′-GATGACTTGGGCAAAGGTGG-3′, R, 5′-CTGCGCATCCCATCACTC-3′; HNF1α: F, 5′-GCCCCTTCATGGCAACCA-3′, R, 5′-CTCTCCCAGGCCAACGT-3′; Sp1: F, 5′-TATGTTGTGGCTGCTACC-3′, R, 5′-TGTGGGATTACTTGATACTGAA-3′; GRHPR: F, 5′-AATTCGGATGACCCCATCC-3′, R, 5′-TCAGGACACCTGGCGTGTAG-3′; CLSTN3: F, 5′-GGACAAGGCAACGGGTGAA-3′, R, 5′-GCCACAGTCATAAGCCTGAATG-3′; and β-actin: F, 5′-ATCCTCTTCCTCCCTGGA-3′, R, 5′-TTCATGGATGCCACAGGA-3′) and quantified with Touch Real-Time PCR detection system (Bio-Rad). Threshold cycle output values for SVCT1, SVCT2, GPX1, SOD1, HNF1α, Sp1, GRHPR, and CLSTN3 mRNA expression levels were normalized relative to β-actin as outlined previously [12,18].

### 2.6. Heterogeneous Nuclear RNA (hnRNA) Analysis

Mouse SVCT1 and SVCT2 hnRNA expression in jejunum was quantified by RT-qPCR using cDNA prepared from 5xFAD and WT littermate mice jejunum mucosa RNA samples (6–8 animals/group) and appropriate hnRNA primers (SVCT1: F, 5′-GCTTCCAGGCTCTAGATGGT-3′, R, 5′-GGGCAAAATCTTCGTTGGGT-3′; SVCT2: F, 5′-ACTCTTGTCCATGGCTCTGG-3′, R, 5′-GGGCAAAATCTTCGTTGGGT-3′; and β-actin: F, 5′-AGATGACCCAGGTCAGTATC-3′, R, 5′-GAGCAGAAACTGCAAAGAT-3′) that anneal to sequence within the intron region. The protocol used for RT-qPCR analysis has been previously established [12,18]. 

### 2.7. Western Blot Analysis

The soluble protein fractions from 5xFAD and WT littermate mice jejunum mucosa and hippocampus samples (4–7 animals/group) were separated using radioimmunoprecipitation (RIPA) buffer (Cat# R0278; Sigma) containing protease inhibitor cocktail (Cat# PI78410; Roche, NJ, USA) by homogenization, sonication and centrifugation at 14000 rpm for 20 min at 4°C. Bio-Rad DC Protein Assay reagents were used in determining the protein concentrations. Sixty micrograms of protein was loaded into an Invitrogen NuPAGE 4–12% Bis-Tris mini gel (Cat# NP0321; Invitrogen) for western analysis. The separated proteins were then transferred to an Immobilon polyvinylidene difluoride (PVDF) membrane (Millipore, Burlington, MA) by electroblotting [18,19]. The membrane was blocked using blocking buffer (Cat# 924-40010; LI-COR Biosciences) then probed with well characterized primary antibodies (raised by Thermo Fisher Scientific, Rockford, IL, USA) against SVCT1 (amino acids: 576-594 RGFSKKTQNQPPVLEDTPD), or SVCT2 (amino acids: 627-645 GYTWKGLRKSDNSRSSDED) (1:500 dilution for both [19]) and/or β-actin (1:2000 dilution) antibodies (Santa Cruz Biotechnology). LI-COR anti-rabbit IRDye 800 (Cat# NC9401842) and/or anti-mouse IRDye 680 (Cat# NC0046410) (1:30000 dilution for both) were used as secondary antibodies to probe the blot at room temperature (RT) for 45 min. Visualization of immunoreactive fluorescent bands was achieved via a LI-COR Odyssey infrared imager and accompanying software, which was also used to quantify specific band intensity.

### 2.8. Immunohistochemistry

Paraformaldehyde-fixed mice hippocampus samples were stored at -20°C in cryo buffer (0.02% NaN_3_, 30% ethylene glycol, 30% glycerol, and 40% 1X PBS at pH 7.4). Tissues were dehydrated for 2 h in a 10% sucrose + 0.02% NaN_3_ + 1X PBS pH 7.4 solution, followed by 12 h in 20% sucrose, and followed by another 12 h in 30% sucrose before cryo-sectioning. After dehydration, hippocampi were embedded in Leica Surgipath FSC22 (Cat# 3801480) and sectioned at a 30μm thickness (coronal) using a Microm HM525NX Cryostat (Thermo Fisher Scientific) at –20°C. Sections were stored in a 24-well plate with 0.02% NaN_3_ + 1X PBS prior to staining. Representative coronal sections (3-4 sections/brain, 4 brains/group) through the caudal diencephalon were selected for staining and were washed three times at 5 min each in 0.3% Tween-20 + 1X TBS. Tissues were blocked for 30 min at RT in 1X TBS buffer (containing 4% BSA + 0.1% Tween-20) then probed with well characterized custom made rabbit anti-SVCT2 primary antibody (1:1000 dilution; Thermo Fisher Scientific [19]) in 1X TBS buffer (containing 0.1% Tween-20 + 0.2% BSA) for 1 h at RT, then overnight at 4°C. Sections were then washed three more times, and then stained with secondary antibody Donkey Anti-Rabbit Alexa Flour 568 (Cat# ab175470; Abcam, MA) in 1X TBS buffer (0.1% Tween-20 + 4% BSA) for 1 h at RT. We tested tissue by omission of the primary antibody from the protocol to confirm the absence of any non-specific binding of secondary antibody to the mouse tissue. No immunofluorescence was detected after anti-rabbit secondary antibody incubation. Tissues were washed three more times and stained with 1X DAPI (Cat# 62248; Thermo Fisher) in TBS for 10 min and mounted on microscope slides with cover slips and SlowFade^TM^ Gold Antifade Mountant media (Cat# S36937; Invitrogen). Stained sections were imaged on an Olympus FV3000 laser-scanning confocal microscope with 40x objective lens to obtain 25–30µm z-stacks. Three-dimensional (3D) algorithm-based volumetric quantification of immunoreactive puncta was facilitated by the AutoQuant (Media Cybernetics) and Imaris modules (version 9.0, BitPlane, Inc., Switzerland) as described [20].

### 2.9. Statistical Analysis

AA uptake data are a percentage of means ± SE of samples from at least 3 mice. The carrier-mediated process was assessed as previously described [12,18]. Data were subjected to Student’s *t*-test with *p* value of 0.05 or less being considered significant. Colorimetric assay, western blot, RT-qPCR and immunohistochemistry data presented are resultant of three or more sample preparations using at least three sets of mice. 

## 3. Results

### 3.1. Level of AA in 5xFAD Mice Serum Samples

**Figure 1:**
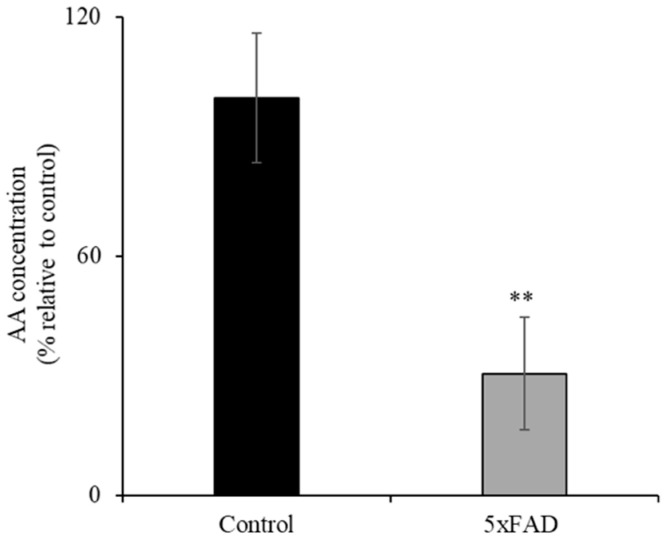
Decreased level of ascorbic acid (AA) in the serum of transgenic mice expressing five Familial Alzheimer’s Disease (5xFAD) mutations. AA levels were determined in 12-month-old 5xFAD and wild-type (WT) littermate mice serum using the Colorimetric Assay Kit II as described in the “Methods”. Values are means ± SE of at least four different mice (*n* = 4). ** *p* < 0.05.

AD patients have been reported to have sub-optimal AA levels [6]. Here we assessed whether the 5xFAD mouse model of AD also displays decreased level of AA. To compare the levels of ascorbate in the circulation of 12-month-old 5xFAD and WT littermate mice, we used a colorimetric method [21,22] (Figure 1). The concentration of AA in the serum of 5xFAD mice was significantly reduced (*p* < 0.05) compared to WT littermates. These data clearly demonstrate depleted vitamin C in the blood circulation in this AD mouse model.

### 3.2. Levels of Oxidative Stress Response Genes Expression in the Intestine of the 5xFAD Mice

**Figure 2:**
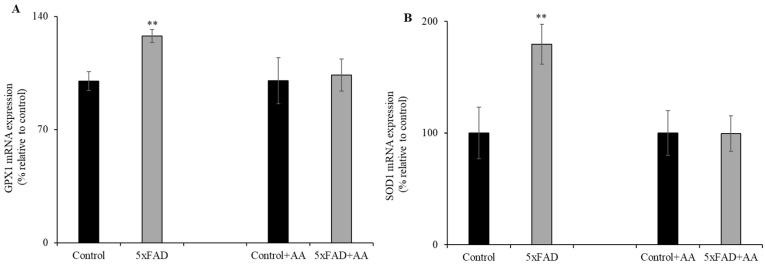
Levels of oxidative stress response genes expression in 5xFAD mice jejunum mucosa. RT-qPCR from reverse-transcribed total RNA of 5xFAD and WT littermate mice (maintained with and without vitamin C supplementation for 1 week) jejunum mucosal scraping was utilized to determine the mRNA expression levels of glutathione peroxidase 1 (GPX1) (A) and superoxide dismutase 1 (SOD1) (B). Values are means ± SE of at least four different mice (*n* = 4). ** *p* < 0.05.

Previous data have shown that vitamin C deficiency leads to oxidative stress in various tissues [23,24,25]. Therefore, we examined the effect of the chronically depleted vitamin C levels (Figure 1) on intestinal expression of oxidative stress response genes. Levels of glutathione peroxidase 1 (GPX1) and superoxide dismutase 1 (SOD1) in 5xFAD mice jejunum mucosa were assessed by RT-qPCR (Figure 2A,B). mRNA levels for both genes were significantly (*p* < 0.05) increased in the jejunum of 5xFAD compared to WT littermate mice. In another study, we aimed to determine whether AA supplementation would reduce the oxidative stress response genes expression in the 5xFAD (which undergoes continuous oxidative stress [26]) mice jejunum mucosa. To do this, we fed 5xFAD mice and WT littermates with AA (3.3g/L) [22,27] for 1 week. Interestingly, we found that the upregulation of GPX1 and SOD1 in the diseased mice were corrected by vitamin C supplementation (Figure 2A,B). These findings suggest enhanced oxidative stress in the jejunum of AD mice that is correctable by vitamin C supplementation. 

### 3.3. ^14^C-AA Uptake Upregulated in the Jejunum of 5xFAD Mice

**Figure 3:**
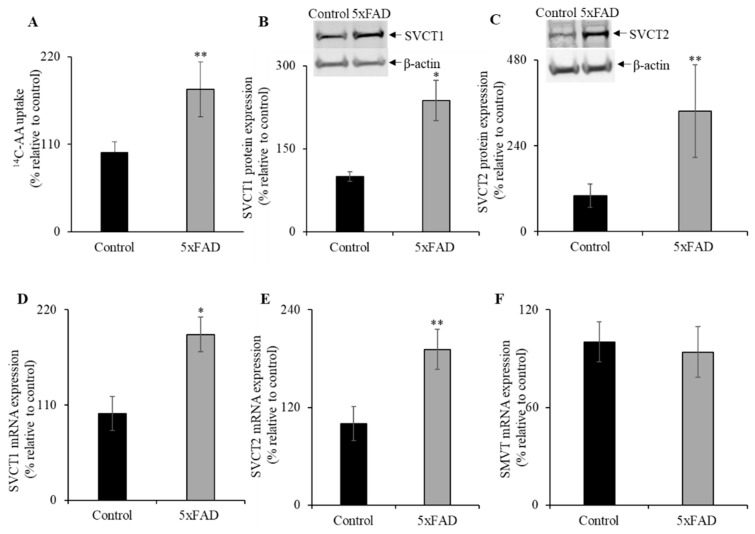
Upregulation of vitamin C transporters functional expression in 5xFAD mice jejunum. (A) Mouse jejunum segments were prepared from 5xFAD and WT littermate mice as described in “Methods”. We performed carrier-mediated ^14^C-AA (0.1μCi) uptake in jejunum segments. (**B**,**C**) Mouse jejunum mucosal protein (60 µg) was isolated from 5xFAD and WT littermate mice and used for western blot analysis. Jejunum protein was separated on NuPAGE 4–12% mini gel and proteins were transferred onto polyvinylidene difluoride (PVDF) membrane. Blots were incubated with custom-made anti-sodium-dependent vitamin C transporter 1 (SVCT1) and anti-SVCT2 rabbit polyclonal and anti-β-actin monoclonal primary antibodies. The specific bands were detected using LI-COR imaging system. (**D**–**F**) RT-qPCR was performed using cDNA prepared from 5xFAD and WT littermate mice jejunum mucosa, and the mouse SVCT1, SVCT2, and sodium-dependent multivitamin transporter (SMVT) primers and normalized relative to simultaneously amplified β-actin. Data are means ± SE of at least four (*n* = 4–8) mice. * *p* < 0.01; ** *p* < 0.05.

Studies have shown that the nutrient transport, including water-soluble vitamins, is upregulated during nutrient deficiency [10,28,29,30,31,32,33]. Therefore, we determined AA uptake in the intestine of 5xFAD and WT littermate mice jejunum [12,18] (Figure 3A). A significant (*p* < 0.05) increase in AA uptake was observed in 5xFAD mice jejunum compared to WT littermates. These data suggest that the AA uptake in the AD intestine is compensatory/adaptively upregulated in response to vitamin C depletion.

### 3.4. SVCT1 and SVCT2 Protein and mRNA Expression Levels in the Jejunum of 5xFAD Mice 

Next, we examined the protein and mRNA expression levels for SVCT1 and SVCT2 by western blot and RT-qPCR, respectively (Figure 3B,C). Both SVCT1 and SVCT2 protein expression levels were markedly increased in the 5xFAD mice jejunum compared to WT littermates. In addition, both SVCT1 and SVCT2 mRNA expression levels were significantly increased in 5xFAD compared to WT littermates (*p* < 0.01 and *p* < 0.05 for SVCT1 and SVCT2, respectively; Figure 3D,E). In contrast, we did not find a marked change in the mRNA levels for the SMVT (Figure 3F), a sodium-dependent multivitamin transporter localized at the apical membrane domain of enterocytes that transports another water-soluble vitamin, biotin [34]. These findings suggest that the upregulation of vitamin C transport system in the AD mice jejunum is substrate-specific.

### 3.5. Transcriptional Upregulation of SVCT1 and SVCT2 in the Jejunum of 5xFAD Mice

**Figure 4:**
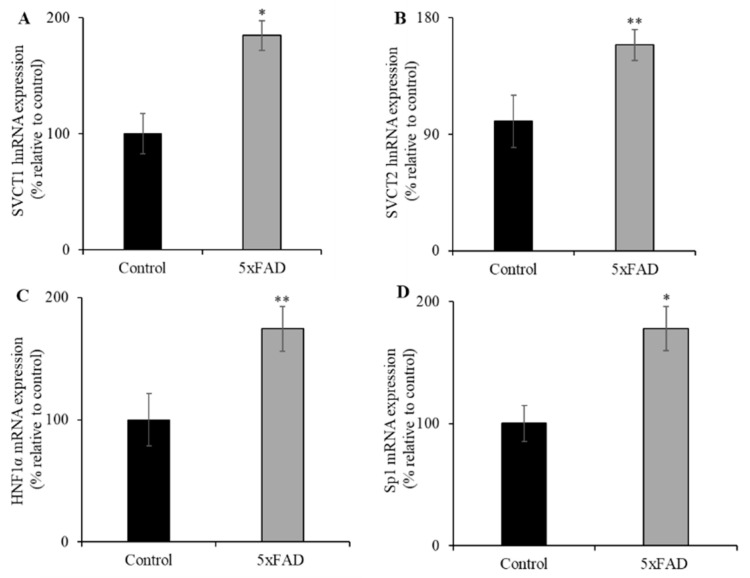
Increased levels of expression of heterogeneous nuclear RNA (hnRNA) and nuclear factors (HNF1α and Sp1) in 5xFAD mice jejunum mucosa. (**A**–**D**) RT-qPCR was performed using cDNA prepared from 5xFAD and WT littermate mice jejunum mucosa, and the mouse SVCT1 and SVCT2 hnRNA primers, and normalized relative to simultaneously amplified β-actin, as well as HNF1α and specific protein 1 (Sp1) mRNA primers that were normalized relative to simultaneously amplified β-actin. Values are means ± SE of at least six mice (*n* = 6) samples. * *p* < 0.01; ** *p* < 0.05.

Changes in mRNA expression levels of the vitamin C transporters could occur through changes in transcription rate or due to involvement of the expression of transcriptional factor(s) of the relevant genes (i.e., *Slc23a1* and *Slc23a2* genes). To examine the first possibility that the rate of transcription of both *Slc23a1* and *Slc23a2* genes in 5xFAD mice might be altered, the expression level of SVCT1 (*Slc23a1*) and SVCT2 (*Slc23a2*) heterogenous nuclear RNA (hnRNA, the first product of gene transcription, and its expression level reflects transcriptional activity of a given gene [35]) was quantified by RT-qPCR using gene specific primers (Figure 4A,B). The hnRNA expression levels were significantly increased for both SVCT1 (*p* < 0.01) and SVCT2 (*p* < 0.05) in 5xFAD mice jejunum compared to WT littermates. To test the second possibility of involvement of transcriptional factor(s), we determined the expression levels of hepatocyte nuclear factor 1 alpha (HNF1α) and specific protein 1 (Sp1) in jejunum of 5xFAD and WT littermate mice. HNF1α and Sp1 are known to regulate the *SLC23A1* (SVCT1) and *SLC23A2* (SVCT2) basal promoter activity in many cellular systems, respectively [36,37,38]. The results showed that HNF1α and Sp1 mRNA expression levels were markedly increased in the jejunum of 5xFAD mice compared to WT littermates (Figure 4C,D). These results suggest that the upregulation under vitamin C depleted conditions is regulated in part by transcriptional mechanism(s).

### 3.6. Expression of GRHPR and CLSTN3 mRNA in Jejunum of 5xFAD Mice 

**Figure 5:**
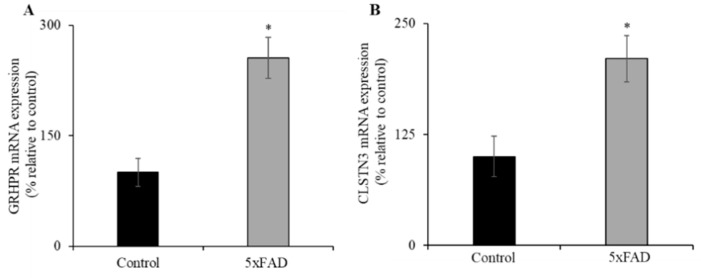
Increased levels of expression of vitamin C transporters interacting protein partners (glyoxylate reductase/hydroxypyruvate reductase (GRHPR) and Calsyntenin 3 (CLSTN3)) in 5xFAD mice jejunum mucosa. (**A**,**B**) RT-qPCR was done using cDNA prepared from 5xFAD and WT littermate mice jejunum mucosa, and GRHPR and CLSTN3 primers, and normalized relative to simultaneously amplified β-actin. Values are means ± SE of at least seven mice (*n* = 7–8) samples. * *p* < 0.01.

Recently, we identified glyoxylate reductase/hydroxypyruvate reductase (GRHPR) and Calsyntenin 3 (CLSTN3) as interacting protein partners of SVCT1 and SVCT2, respectively; these interactions enhance functional expression of the SVCT1 [39] and SVCT2 (unpublished observations). Both GRHPR and CLSTN3 mRNA expression levels were significantly increased (*p* < 0.01 for both) in the jejunum of 5xFAD mice compared to WT littermates (Figure 5A,B). These findings suggest that GRHPR and CLSTN3 may also contribute to the upregulation of AA uptake in the jejunum of 5xFAD mice. 

### 3.7. Hippocampal Expression of SVCT2 in the 5xFAD Mice

**Figure 6:**
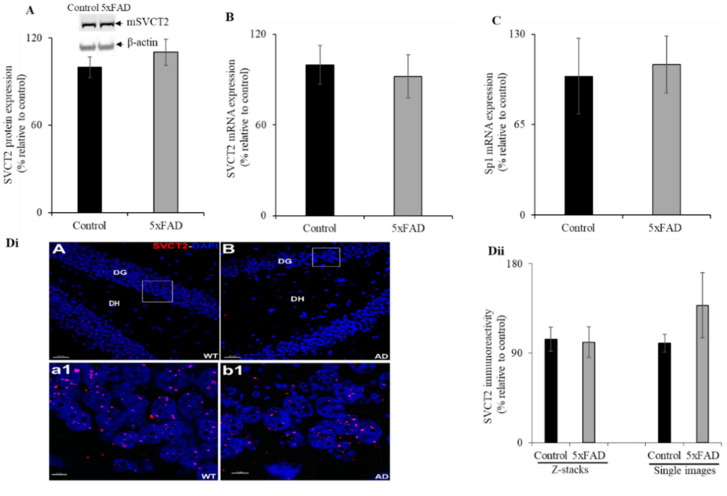
Expression levels of mSVCT2 protein, mRNA, and nuclear factor (Sp1) in 5xFAD mice hippocampus. (**A**) Western blot was performed using hippocampus protein (60 µg) samples isolated from 5xFAD and WT littermates as described in “Methods”. The blots were probed with mouse SVCT2 antibodies. (**B**,**C**) RT-qPCR was done using SVCT2 or Sp1 primers and cDNA synthesized from mouse hippocampus RNA samples. (**Di**) Immunohistochemistry was performed using 5xFAD and WT littermate mouse hippocampus sections and SVCT2 antibody as described in “Methods” and representative low-magnification image displayed (Di_A-B_, DG—dentate gyrus, DH—dentate hilus; these are hippocampal sub-regions; scale bar 30 μm). The highlighted (white box) regions of the 5xFAD (Alzheimer’s disease, AD) and WT were the high-magnification images, respectively (Di_a1-b1_, scale bar 5 μm). (**Dii**) Volumetric quantification of the SVCT2 immunoreactive puncta in the 5xFAD and WT littermates. Values are means ± SE of at least five mice (*n* = 5–12) samples.

Given that AD is a disease of the brain and its primary region of neurological impact is the hippocampus, we aimed to determine the status of SVCT2 (SVCT2 is mainly expressed in brain [9,14]) protein and mRNA expression in the 5XFAD mouse hippocampus. Western blot analysis showed no significant difference between SVCT2 protein expression levels in the hippocampus of 5xFAD mice compared to WT littermates (Figure 6A). Similarly, there was no difference in SVCT2 mRNA expression observed between the hippocampus of 5xFAD and WT littermate mice (Figure 6B). Further, we also determined no difference in Sp1 mRNA expression between 5xFAD and WT littermate mice (Figure 6C). Immunohistochemistry with high-magnification images for each group showed SVCT2 puncta within the granule cell layer of the dentate gyrus (Figure 6Dii). We found a similar but sparse distribution of SVCT2 immunoreactivity throughout the hippocampal sub-regions. Such differences with Marcos et al. [40] arise due to differences in the staining procedures including incubation with detergents like Tween-20 and inclusion of the blocking serum in primary and secondary antibody staining solutions in order to reduce non-specific binding. The SVCT2 immunoreactive puncta in the mice hippocampus using volumetric quantification [20] did not reveal significant differences between the 5xFAD mice compared to WT littermates (Figure 6Dii). 

## 4. Discussion

Clinical observations have documented reduced blood plasma vitamin C levels in AD patients compared to age-matched healthy controls [2,8,9,41,42]. It has been well established that transport of a variety of nutrients including water-soluble vitamins in the intestine and other tissues is adaptively regulated by their levels in the diet as well as in blood circulation [10,28,29,30,31,32,33,43]. Currently, not much research exists to implicate an effect of vitamin C depleted conditions in the intestinal and brain vitamin C transport process in AD. Further research is needed as deficiency of vitamin C plays an important role in cognitive dysfunction and accelerates β-amyloid accumulation and deposition in AD brain [3,5,6,44]. Previous studies have shown that consumption of vitamin C in the AD brain is high owing to their oxidative stress, which may lead to the depletion of essential micronutrient/antioxidant vitamin C levels in the blood circulation [6,45,46]. In addition, studies have shown lower levels of plasma vitamin C in AD patients despite normal dietary intake [42,46].

It is well established that vitamin C deficiency/insufficiency leads to increased oxidative stress and induces the oxidative stress response markers activity in different tissues [23,24,25]. Similarly, our study also shows vitamin C depletion as well as elevated levels of oxidative stress markers in the jejunum of 5xFAD mice compared to WT littermate controls. This increase in oxidative response genes expression levels can be explained as a possible means to induce a compensatory/adaptive regulatory mechanism(s) to tolerate oxidative damage in the oxidative stress conditions that occur in AD. Interestingly, the 5xFAD mice supplemented with vitamin C showed markedly reduced levels of expression of oxidative stress response genes, which indicates that increased bioavailability of vitamin C in the intestine reduces the oxidative stress in these animals to overcome further deterioration from AD pathology as shown before [27]. Based on these findings, future studies can explore possible therapeutic approaches (i.e., different formulation and mode of administration of vitamin C) to increase the capability of intestinal vitamin C absorption or bioavailability of vitamin C to enterocytes as a means of restoring normal levels of vitamin C in AD.

In a previous in vitro model, vitamin C deficiency caused upregulation of vitamin C uptake and associated increased expression of its transporters [37]. However, in the current investigation, the observed upregulation of AA uptake and increased expression levels of both SVCT1 and SVCT2 in the jejunum of 5xFAD mice compared to WT littermates suggest that these animals were undergoing oxidative stress owing to their AD pathology and, consequently, depleting vitamin C levels in this important organ triggered a compensatory response. The observed changes in SVCT1 (*Slc23a1*) and SVCT2 (*Slc23a2*) mRNA expression levels could occur via changes in transcription rate or due to the involvement of expression of nuclear factors of the respective genes. The transcriptional rate (hnRNA expression levels) of both *Slc23a1* and *Slc23a2* genes were determined to be upregulated, which is similar to the results of previous studies on vitamin B transporter genes expression under its deficient condition [33]. In addition, the nuclear factors, HNF1α and Sp1, expression levels were also upregulated in the jejunum of 5xFAD mice. It is interesting to mention here that HNF1α and Sp1 were upregulated under vitamin C and riboflavin deficiency, respectively, in different cell culture models [33,37]. Together these findings further support a role for both nuclear factors in the upregulatory effect in the jejunum of 5xFAD mice. Furthermore, the recently identified novel interacting protein partners GRHPR (SVCT1) [38] and CLSTN3 (SVCT2) (unpublished observations) were also induced in the jejunum of 5xFAD mice, suggesting that both interactors are also playing a role in the compensatory/adaptive regulatory mechanism(s) in AD pathology.

Next, we observed no significant change in the SVCT2 expression in the hippocampus of 5xFAD mice. This is possible due to the characteristically high vitamin C concentration in the brain and also because the hippocampus has the highest vitamin C content than any other region of the brain [5,47,48,49]. Previous studies have shown high SVCT2 mRNA [50] and protein [51] expression in the hippocampus. Further, Qiu et al. [52] described the importance of SVCT2 for the normal neuronal growth and reduced oxidative damage in the hippocampus. Recently, Dixit et al. [4] showed increased accumulation of amyloid β-plaques in hippocampus of *Slc23a2^+/-^* APP/PSEN1^+^ transgenic mice compared to APP/PSEN1^+^ mice with normal vitamin C level in the brain indicating the critical roles of vitamin C transporter in the neuronal system. During vitamin C insufficiency, the brain may be able to retain tenaciously more vitamin C because of the high level of SVCT2 activity. Whether this phenomenon is specific to the 5xFAD accelerated mice model of AD requires further investigation using other transgenic AD mice models, such as J20 and 3xTg, as well as human data.

In summary, our findings demonstrate for the first time that vitamin C is depleted in an AD mouse model and that there is a compensatory increase in AA uptake and SVCT1 and SVCT2 transporter expression in the jejunum of 5xFAD mice. This compensatory response is mediated at least in part via transcriptional regulation of both the involved *Slc23a1* and *Slc23a2* genes.

## Data Availability

The data presented in this study are available within the article.
